# A Rare Occurrence of Ectopic Parathyroid Carcinoma Presenting as a Case of Recurrent Fractures

**DOI:** 10.7759/cureus.51404

**Published:** 2023-12-31

**Authors:** Saad Bin Zafar Mahmood, Aisha Jamal, Zain Mushtaq, Muhammad Q Masood

**Affiliations:** 1 Medicine, Aga Khan University Hospital, Karachi, PAK; 2 Internal Medicine, Aga Khan University Hospital, Karachi, PAK; 3 Medicine, Endocrinology, Aga Khan University Hospital, Karachi, PAK

**Keywords:** end-stage renal disease (esrd), skeletal fractures, hypercalcemia, ectopic parathyroid carcinoma, primary hyperparathyroidism

## Abstract

Ectopic parathyroid tumors are uncommon, accounting for only 6% of parathyroid adenomas, and even fewer cases are attributed to parathyroid carcinomas. While ectopic parathyroid carcinoma in the anterior mediastinum is a rare condition, the occurrence of recurrent skeletal fractures in the presence of mild hypercalcemia is even rarer. In this report, we present the case of a 52-year-old man with a medical history of hypertension, chronic kidney disease, and a previous right-sided intertrochanteric fracture of the femur one year earlier. He presented to the emergency room with left-sided hip pain and shortness of breath due to volume overload. Laboratory tests revealed mild hypercalcemia and hyperparathyroidism, with normal vitamin D levels. An X-ray showed a left neck of femur fracture. Further evaluation with a three-phase skeletal scintigraphy indicated the presence of metabolic bone disease. A contrast-enhanced computed tomography of the chest revealed a solitary soft tissue nodule in the anterior mediastinum, representing an ectopic parathyroid adenoma. The lesion was successfully removed using video-assisted thoracic surgery, and the histopathological analysis confirmed the diagnosis of parathyroid carcinoma. The combination of mild hypercalcemia and recurrent fractures is an unusual presentation of ectopic parathyroid carcinoma, underscoring the importance of considering this condition as a potential cause in similar cases to ensure timely and appropriate treatment.

## Introduction

Primary hyperparathyroidism (HPT) is considered to be the most common cause of hypercalcemia, with parathyroid adenomas (nearly 80% of cases) forming the bulk of these cases [1]. Parathyroid hyperplasia (10% to 15% of cases) and, infrequently, parathyroid carcinomas (<1% to 5% of cases) can also lead to HPT [2]. Parathyroid carcinomas are an unusual cause of hypercalcemia, leading to poor outcomes, and are preferably managed by surgical resection [3]. Ectopic parathyroid tumors are rare, with only 6% of parathyroid adenomas attributed to ectopic growth and even fewer attributed to parathyroid carcinomas [3,4]. We present the case of a 52-year-old male who presented with hypercalcemia and recurrent pathological fractures and was found to have a parathyroid carcinoma.

## Case presentation

A 52-year-old man with a prior history of hypertension, chronic kidney disease (CKD), and a right-sided intertrochanteric (IT) fracture one year ago presented to the emergency department with complaints of left-sided hip pain for the past two weeks. His hypertension and CKD (baseline creatinine between 2.0-2.5 mg/dL) were otherwise stable but he complained of intermittent dyspnea.

**Figure 1:**
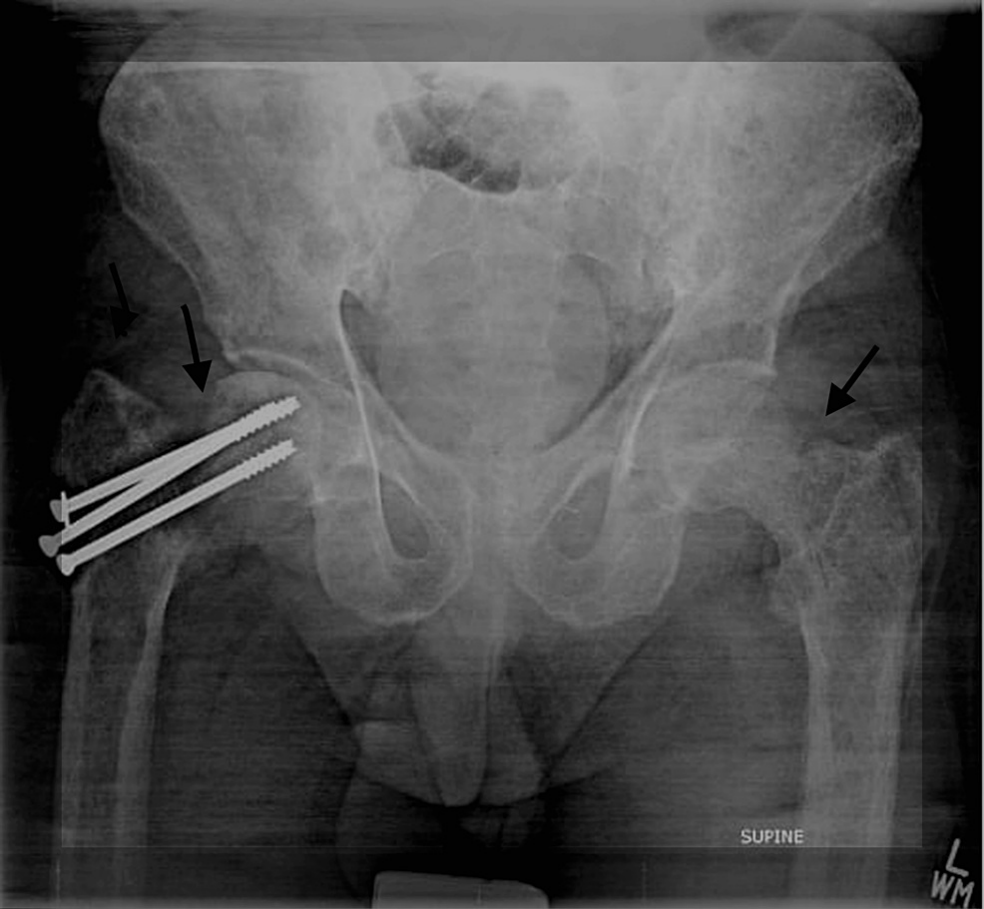
X-ray pelvis anteroposterior view. Visualized bones show a significantly reduced bone density. Suspicion of undisplaced left femoral neck fracture.

On examination, he had a blood pressure of 110/70 mmHg, respiratory rate of 14 breaths per minute, heart rate of 68 beats per minute, and was afebrile. He was unable to move his left leg due to pain. The rest of the systemic examination was unremarkable apart from bibasilar crepitation suggesting volume overload. Initial laboratory investigations showed thrombocytopenia (platelets 140 x 109/L), with hemoglobin of 12.4 g/dL, total leukocyte count of 6.9 x 109/L, and erythrocyte sedimentation rate of 21 mm/first hour. He had an elevated creatinine level of 3.2 mg/dL, with hypokalemia (2.7 mmol/L), hypercalcemia (corrected calcium of 12.1 mg/dL), and hyperphosphatemia (5.2 mg/dL). The rest of the investigations were normal. The patient had no complaints of constipation, abdominal pain, increased urinary frequency, or muscle weakness. A pelvis X-ray revealed an undisplaced fracture of the left femoral neck (Figure 1). He was initially managed with intravenous hydration and analgesics.

Keeping in view the history of repeated pathological fractures, a three-phase skeletal scintigraphy was ordered which was suggestive of metabolic bone disease. Further workup was pursued to rule out causes of pathological fractures. (Table 1) He was found to have hyperparathyroidism (parathyroid hormone (PTH) level 1,347 pg/mL) with a normal 25 hydroxy vitamin D (23.6 ng/mL). Ultrasound of the neck showed a normal thyroid with a small nodule in the lower pole of the right lobe. Ultrasound of the kidneys showed echogenic kidneys bilaterally with altered corticomedullary differentiation suggestive of bilateral renal parenchymal disease.

**Table 1:** Laboratory investigations.

Laboratory parameters	Reference range	Lab values
Serum prostate-specific antigen (ng/mL)	0–3.5	1.05
Erythrocyte sedimentation rate (mm/first hour)	0–15	21
Serum 25-hydroxy vitamin D (ng/mL)	>30	23.6
*Brucella *antibody	<1:80	<1:80
Serum calcium (mg/dL)	8.6–10.2	10.8
Serum albumin (g/dL)	3.5–5.2	3.7
Plasma parathyroid levels (pg/mL)	16–87	1347
Serum phosphate (mg/dL)	2.5–4.5	5.2
Serum alkaline phosphatase (g/dL)	45–129	1,134

**Figure 2:**
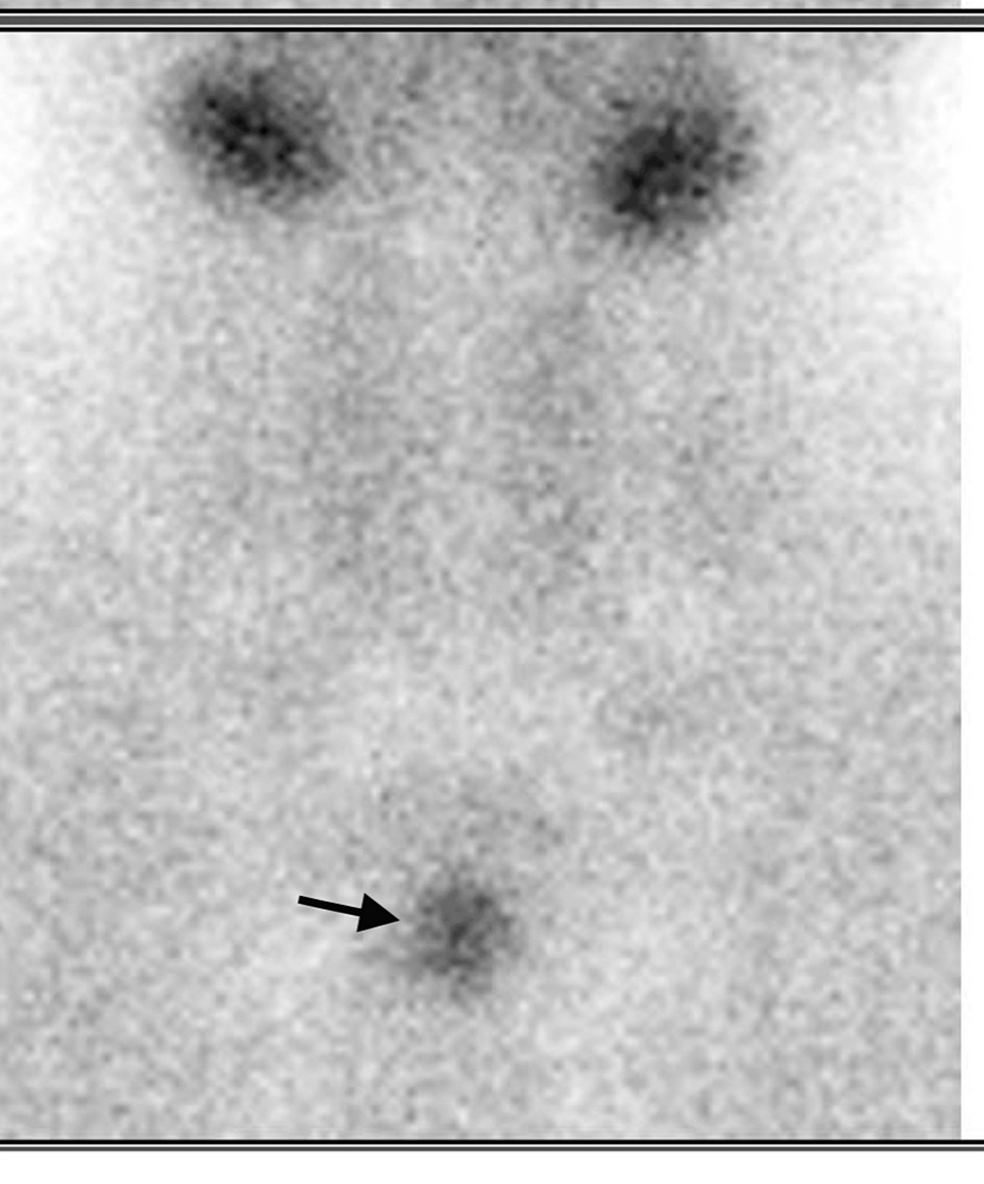
Parathyroid scintigraphy with 622 MBq of Tc-99m sestamibi injected intravenously. Solitary parathyroid adenoma posterior to manubrium sterni (ectopic position).

**Figure 3:**
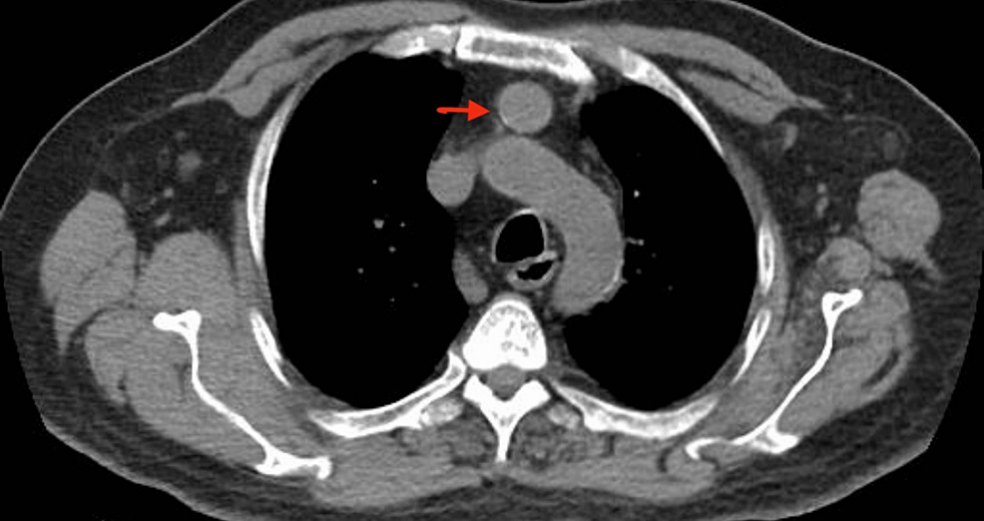
CT of the chest without contrast showing a well-circumscribed soft tissue nodule in the anterior mediastinum.

**Figure 4:**
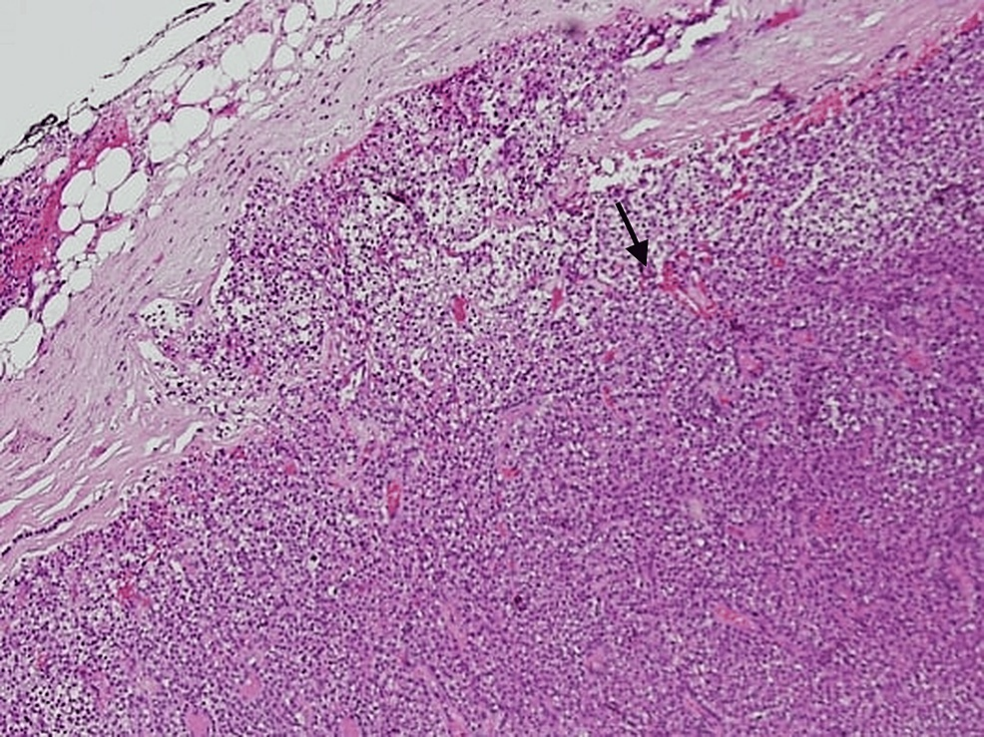
Histopathology showing an encapsulated, circumscribed neoplasm in the parathyroid gland. It is composed of a mixture of eosinophilic and pale cells, with predominantly round, bland nuclei. Additionally, there are areas showing random nuclear atypia, along with foci of fibrocollagenous septae. Findings are suggestive of parathyroid carcinoma.

Further radiographic imaging studies including parathyroid scintigraphy and computed tomography (CT) of the chest (without contrast due to raised creatinine) were performed for localization of a lesion which showed a solitary soft tissue nodule in the anterior mediastinum representing ectopic parathyroid adenoma (Figures 2, 3). The patient underwent video-assisted thoracoscopic (VATS)-guided removal of parathyroid tissue. Histopathology later revealed it to be a parathyroid carcinoma (Figure 4).

Postoperatively, the patient remained in the intensive care unit and developed worsening renal functions for which he required hemodialysis; however, other than that he made an uneventful recovery. There were no signs or symptoms of hungry bone syndrome and was subsequently discharged from the hospital. The patient was followed up in the clinic after one month. He had no active complaints, and a summary of his current and previous labs is presented in Table 2.

**Table 2:** Laboratory analysis before and after surgery.

Laboratory parameters	Reference range	Preoperative labs	Postoperative labs (1 month)
Fasting parathyroid levels (pg/mL)	16–87	1,347	902
Serum calcium (mg/dL)	8.6–10.2	10.8	7.8
Serum phosphate (mg/dL)	2.5–4.5	5.2	2.4
Serum magnesium (mg/dL)	1.6–2.6	1.9	2.2
Serum 25-hydroxy vitamin D levels (ng/mL)	>30	23.6	26.7

## Discussion

We report a rare case of mediastinal parathyroid carcinoma presenting with repeated neck of femur fractures. Parathyroid carcinomas are a rare condition, accounting for only 1% of HPT cases which occur equally in males and females, typically presenting at around 45-51 years old [5]. Ectopic parathyroid carcinomas are even rarer, with only 31 cases of ectopic mediastinal parathyroid carcinomas reported to date, mostly in the superior mediastinum [6]. Diagnosis of parathyroid carcinoma is more likely if the patient has high serum calcium levels (≥14 mg/dL), high parathyroid hormone levels (twice the upper limit of normal), and osteitis fibrosa cystica (a bone condition associated with long-standing hyperparathyroidism) [7].

The rarity of our case appears to be the absence of any symptoms related to hypercalcemia and the only presenting complaints of recurrent neck of femur fracture and mild hypercalcemia. Despite having generalized bone pain indicative of osteitis fibrosa cystica, there was no palpable neck lump upon examination. Although a literature review does show that 14.3% of parathyroid carcinoma exhibited skeletal complications, only two other case reports have described ectopic parathyroid carcinomas with presenting features of recurrent fractures [8,9].

Our patient also had asymptomatic nephrolithiasis and an advanced CKD which progressed to end-stage renal disease requiring renal replacement therapy during his illness requiring long-term hemodialysis. Literature reports that approximately 28.6% of parathyroid carcinoma had renal involvement with the most common renal manifestations being nephrolithiasis (and reduced glomerular filtration rate) [10]. To our knowledge, end-stage renal disease, as in our case, has only been reported in three other cases of ectopic parathyroid carcinoma [11-13].

Our patient’s diagnosis was determined by a combination of his clinical and pathological features. A study by Robert et al., which reviewed 311 patients with HPT, including nine with parathyroid carcinomas, found that when PTH levels are less than four times the upper limit of normal and tumor weight is less than 1.9 g, the likelihood of parathyroid carcinoma is zero [14]. Our patient had mild hypercalcemia (12.4 mg/dL), significantly elevated PTH levels (1,347 pg/mL), a tumor weight of 5 g, and a history of recurrent femur fractures.

Imaging plays an important role in distinguishing between benign and malignant parathyroid lesions. Ultrasound is the safest and simplest modality, with a sensitivity of 71% and a specificity of 100% [15]. In contrast, 99mTc-MIBI scintigraphy is the preferred diagnostic test for identifying and localizing parathyroid carcinoma, as noted by studies [16]. A combination of imaging techniques, such as ultrasound, CT scan, and MIBI scintigraphy, has a sensitivity of 100% in identifying parathyroid carcinoma [17]. Our case followed a similar diagnostic algorithm starting with an ultrasound of the neck, followed by a 99mTc-MIBI scintigraphy and a CT scan. Although the location was identified appropriately, the presumed diagnosis before the histopathological diagnosis was of a parathyroid adenoma. The definitive diagnosis of our case was also based on the capsular and lymphovascular invasion. A previous study postulated that the definitive diagnosis of parathyroid carcinoma can be made by invasion of surrounding tissues or distant metastasis. Hence, it is very difficult to diagnose and is mostly confirmed postoperatively on histopathology [18].

Surgical treatment via en bloc resection remains the gold standard treatment for parathyroid carcinoma with a five-year survival rate of 60-93% and hypercalcemia being a major cause of mortality in such patients [18]. Our patient underwent a VATS-guided removal which has been to be a better modality for parathyroid tumor excision in contrast to open surgery in terms of short intraoperative time, bleeding, as well as reduced length of hospital stay and swift recovery [18]. Amer et al., in their case series of seven cases undergoing VATS between 2004 and 2009 also reported similar findings and postulated that VATS should be considered the first-line modality for mediastinal ectopic parathyroid adenoma resection [19]. Our patient followed up in the clinic twice in the next six months and showed remarkable improvement in all laboratory parameters. However, it remains to be seen if these improvements sustain over an extended period.

## Conclusions

We report a rare case with an incidental finding of ectopic mediastinal parathyroid carcinoma which primarily presented with a pathological fracture. Due to the rarity of the disease, these carcinomas can be easily missed; however, early detection with laboratory tests and imaging (MIBI scintigraphy combined with CT scan or MRI) can effectively help physicians diagnose, and early intervention can be curative.
